# Evolutionary barriers to horizontal gene transfer in macrophage-associated *Salmonella*

**DOI:** 10.1093/evlett/qrad020

**Published:** 2023-05-23

**Authors:** Rama P Bhatia, Hande Acar Kirit, Cecil M Lewis, Krithivasan Sankaranarayanan, Jonathan P Bollback

**Affiliations:** Institute of Infection, Veterinary, and Ecological Sciences, Department of Evolution, Ecology, and Behaviour, University of Liverpool, Liverpool, United Kingdom; Institute of Infection, Veterinary, and Ecological Sciences, Department of Evolution, Ecology, and Behaviour, University of Liverpool, Liverpool, United Kingdom; Laboratories of Molecular Anthropology and Microbiome Research (LMAMR), University of Oklahoma, Norman, OK, United States; Laboratories of Molecular Anthropology and Microbiome Research (LMAMR), University of Oklahoma, Norman, OK, United States; Department of Anthropology, University of Oklahoma, Norman, OK, United States; Laboratories of Molecular Anthropology and Microbiome Research (LMAMR), University of Oklahoma, Norman, OK, United States; Department of Microbiology and Plant Biology, University of Oklahoma, Norman, OK, United States; Institute of Infection, Veterinary, and Ecological Sciences, Department of Evolution, Ecology, and Behaviour, University of Liverpool, Liverpool, United Kingdom

**Keywords:** fitness, gene expression, microbes, adaptation

## Abstract

Horizontal gene transfer (HGT) is a powerful evolutionary force facilitating bacterial adaptation and emergence of novel phenotypes. Several factors, including environmental ones, are predicted to restrict HGT, but we lack systematic and experimental data supporting these predictions. Here, we address this gap by measuring the relative fitness of 44 genes horizontally transferred from *Escherichia coli* to *Salmonella enterica* in infection-relevant environments. We estimated the distribution of fitness effects in each environment and identified that dosage-dependent effects across different environments are a significant barrier to HGT. The majority of genes were found to be deleterious. We also found longer genes had stronger negative fitness consequences than shorter ones, showing that gene length was negatively associated with HGT. Furthermore, fitness effects of transferred genes were found to be environmentally dependent. In summary, a substantial fraction of transferred genes had a significant fitness cost on the recipient, with both gene characteristics and the environment acting as evolutionary barriers to HGT.

## Introduction

Horizontal gene transfer (HGT) is the transfer of genetic material between individuals of the same or different species rather than vertically from parent to offspring. HGT plays an important role in bacterial adaptation: For example, acquisition of accessory genes can confer novel phenotypes to bacteria, such as antimicrobial resistance, microbial toxins, and tolerance to heavy metals (Ochman et al., 2000).

Gene acquisition through HGT may exert different fitness effects on the recipient cell, with these effects determining the evolutionary fate of the newly transferred gene. Fitness can be defined as differential reproductive success and is key to understanding bacterial evolution and adaptation (Orr, 2009). A fitness advantage or disadvantage can be considered at different levels of the biological hierarchy (e.g., genes, transposons, plasmids, cells) that are under selection (Agren, 2016; Draghi & Turner, 2006; Eberhard, 1990; Rodriguez-Beltran et al., 2021).

In this study, the transferred genes that on expression significantly increased the relative fitness of the host were said to have a beneficial effect; those that caused a significant decrease in the relative fitness were said to have a deleterious fitness effect and those that did not significantly differ from the “wild type” were classed as neutral.

While genes with a beneficial or neutral effect can persist in bacterial populations, genes with deleterious effects will most likely be purged (Mira et al., 2001; Popa & Dagan, 2011). The quantitative effects of mutations can be summarized as a distribution—the distribution of fitness effects or DFE—which models the frequency of mutations with different strengths of effects (Eyre-Walker & Keightley, 2007).

Many experimental studies have been carried out to determine the DFE of new mutations. To mention a few examples, DFEs have been estimated for *Escherichia coli* mutants generated by random transposon mutagenesis (Elena et al., 1998), spontaneous and induced mutations in diploid yeast cell lines (Wloch et al., 2001), for random mutations in vesicular stomatitis virus (Sanjuan et al., 2004), for amino acid mutations in humans and *Drosophila melanogaster* (Eyre-Walker et al., 2006; Keightley & Eyre-Walker, 2007), and for point mutations in genes (Lebeuf-Taylor et al., 2019). Parametric distributions such as unimodal log-normal and gamma distributions have been widely used to model and infer properties of DFEs (Kousathanas & Keightley, 2013). Although there are differences in DFEs at the species and genomic level, they do exhibit some general properties, such as beneficial mutations are rare, strongly beneficial mutations are exponentially distributed, and the DFE for deleterious/lethal mutations are complex and multimodal (Eyre-Walker & Keightley, 2007).

DFEs are thus an important predictor of the outcome of HGT events, as they provide insights into the evolutionary fate of a horizontally transferred gene determined by its fitness on the recipient cell. Therefore, understanding the role of factors in defining the DFEs of horizontally transferred genes is critical if we wish to understand bacterial evolution. Collectively, we will refer to these factors as evolutionary barriers to HGT.

Several potential evolutionary barriers to HGT have been predicted using computational approaches such as phylogenetic and parametric/compositional analysis (Ravenhall et al., 2015), including those due to

- Gene function: The “complexity hypothesis” suggests that genes involved in information processing, such as those involved in DNA replication, transcription, and translation are less often transferred compared with operational genes, those involved in amino acid synthesis, nucleotide biosynthesis, energy metabolism (Jain et al., 1999; Nakamura et al., 2004; Rivera et al., 1998).
- Gene interactions: The complexity hypothesis originally proposed that the transferability of genes was dependent on their biological functions (Jain et al., 1999), and later suggested that a major factor affecting HGT was instead the number of connections in gene networks (Cohen et al., 2011). The idea is that an increase in gene connectivity can reduce the fitness of the bacterial host by creating a stoichiometric imbalance in the cell (Omer et al., 2010; Wellner & Gophna, 2008) or by improper interactions with their existing partners in the host (Papp et al., 2003).
- Deviation in GC content: The base composition similarity between ancestral genes within a genome aid in distinguishing a horizontally transferred foreign gene exhibiting different characteristics, like GC content and codon usage patterns, in comparison to the ancestral genes. HGT frequencies can be affected by the donor-recipient similarity barrier. Genes acquired from donors of dissimilar GC content are more likely to be targeted by anti-HGT systems (Labrie et al., 2010; Lucchini et al., 2006; Navarre, 2016; Navarre et al., 2006; Tock & Dryden, 2005). For example, the histone-like nucleoid structuring protein (H-NS) in *Salmonella* can silence the horizontally acquired foreign genes with a GC content lower than the recipient genome (Lucchini et al., 2006).
- Deviation in codon usage: An acquired gene has a rich codon usage if it prefers the abundant codons in the recipient genome, whereas one with a bias for rare codons is said to have nonoptimal codon usage (Medrano-Soto et al., 2004). Codon usage bias can affect the translational efficiency and accuracy of the cell (Qian et al. 2012), for example, by leading to misfolded cytotoxic proteins, which can cause aggregation at the cell membranes, thus disrupting the cell integrity and reducing the fitness of the cell (Drummond & Wilke, 2008).
- Gene length: The length of the gene can impose fitness costs at the chromosome, transcript, and protein levels in the host cell. Although extra DNA is unlikely to be costly at the chromosome/replication level, the energy expenditure inflates with transcription and even more with protein synthesis (Lynch & Marinov, 2015). Ribosomal sequestration may limit the production of essential proteins, lowering host fitness and being selected against in a population (Baltrus, 2013).
- Gene dosage/protein dosage: Protein dosage is a potential barrier to HGT, as changes in protein concentrations due to variation in gene copy numbers can cause an imbalance in the stoichiometry of the cell, thereby reducing bacterial fitness (Papp et al., 2003; Park & Zhang, 2012; Sorek et al., 2007).

In addition to the intrinsic characteristics of the genes themselves, the environment can substantially alter the fitness effects of horizontally acquired genes. For example, genes that confer antibiotic resistance are beneficial in the presence of the antibiotic but deleterious in its absence (Melnyk et al., 2015; Roux et al., 2015). In fact, multiple studies have shown that the environment may affect the selective outcomes in evolving bacterial populations (Cochran et al., 1998; Kishony & Leibler, 2003; Remold & Lenski, 2001).

Here, we experimentally test the fitness effects of HGT events, by introducing genes from *E. coli* into *Salmonella* Typhimurium 4/74 in four different infection-relevant environments. *Salmonella* Typhimurium 4/74 is a bacterium that is highly virulent in cattle, pigs, mice, and chickens (Canals et al., 2019; Kroger et al., 2013). *Salmonella* is an intracellular pathogen, and to survive and proliferate within eukaryotic cells, it must adapt to a fluctuating intracellular environment. During the course of evolution, *Salmonella* has acquired virulence-encoded Salmonella Pathogenicity Islands (SPI) through horizontal gene transfer. Among many other pathogenic properties, SPIs have allowed *Salmonella* to hoodwink host immune surveillance and survive in a replicative niche within a modified phagosome in the host macrophages, known as the *Salmonella*-containing vacuole (Hurley et al., 2020). Among the SPIs, the SPI-2 encoding a type III secretion system is essential for systemic virulence and intramacrophage survival (Cirillo et al., 1998; Deiwick & Hensel, 1999). The in vitro conditions in this study, activating SPI-2 and representing cues encountered by *Salmonella* during infection are based on those published previously (Deiwick & Hensel, 1999; Deiwick et al., 1999; Lӧber et al., 2006).

We tested the fitness consequences of the 44 transferred genes in environments that mimic key features of the macrophage environment (Kroger et al., 2013), using PCN (phosphate/carbon/nitrogen) medium that induces SPI-2 expression (Lӧber et al., 2006; Xu & Hensel, 2010) as the base and further modified it to either contain low magnesium, low oxygen (hypoxic), or an antibiotic challenge using a fluoroquinolone class of drugs—ciprofloxacin, a first-line antimicrobial for many Gram-negative pathogens, including *Salmonella* (see Supplementary Table 1).

In contrast to a retrospective computational approach, our experimental approach allows us to test which genes are likely to fail to survive the evolutionary sieve and thus disentangle the influence of different evolutionary barriers and the role of the environment.

## Materials and methods

### Bacterial strains and growth conditions

*Escherichia coli* K-12 MG1655 and *Salmonella enterica* subsp. *enterica* serovar Typhimurium strain 4/74 (Hoiseth & Stocker, 1981) were used as the donor and recipient strain, respectively. LB Broth (Lennox) and PCN medium (InSPI2 pH 5.8), which induces SPI-2 expression (Lӧber et al., 2006; Xu & Hensel, 2010), were used for growing strains.

### Construction of pZS4-kan-tetR plasmid

Acar Kirit et al. (2020) modified the original pZS4Int plasmid (Lutz & Bujard, 1997) by removing the lacI gene. The modified pZS4Int plasmid contained the tetR gene under the constitutive P_N25_ promoter, spectinomycin resistance gene, and origin of replication pSC101 (Acar Kirit et al., 2020). Since the 4/74 strain exhibited growth in spectinomycin concentrations ranging from 80 to 200 µg/mL, it was replaced with a kanamycin gene using the NEBuilder HiFi DNA Assembly Reaction Protocol.

### Chromosomal modification of *S.* Typhimurium strain 4/74

The construct (tetR gene under the constitutive P_N25_ promoter and kanamycin resistance gene) from the pZS4-kan-tetR plasmid was integrated into the lambda phage attachment site of 4/74 chromosome (see Supplementary Figure 1) using λ red recombination plasmid pSIM5-tet (Datsenko & Wanner, 2000; Koskiniemi et al., 2011). The integration site in the modified chromosome of 4/74 strain was Sanger sequenced to ensure that no mutations were introduced during the recombineering process.

### Genes under study

The genes of interest to be transferred were selected from previous studies (Acar Kirit et al., 2020, 2022). *Escherichia coli* strain K-12 substr. MG1655 (GenBank: U00096.2) was used as the donor. Since the study wanted to understand the role of the barriers affecting HGT, genes that are known to be mobile (e.g., phage-related proteins, insertion sequences, transposable elements) were excluded when selecting genes for transfer. The genes were filtered based on a decision tree and were then selected using an arbitrary selection method with some constraints as follows. The PPI and functional modules for *E. coli* genes were obtained from a previous study (Hu et al., 2009). Since a random selection would be biased toward an overrepresentation of genes contained within large functional modules, sampling of the genes ensured that the genes belonged to different functional modules. Additionally, only genes with validated interactions based on LCMS and MALDI after SPA tagging (Hu et al., 2009) were retained, ensuring that the genes were sampled to include the widest possible representative range of interactions. However, the selected genes did not account for a representative range of metabolic and regulatory interacting partners. Furthermore, sampled genes were not accounted for their origins, resulting in 36 genes being part of the core genome, 5 genes predicted to have been horizontally transferred, and 3 genes with unknown origin.

### Construction of *S.* Typhimurium 4/74 mutant library

The selected 44 *E. coli* orthologs were cloned into a modified version of the pZS* class of plasmids under the control of the p_LtetO-1_ promoter (Lutz & Bujard, 1997), and cloned plasmids were electroporated in *E. coli* in a previous study (see Supplementary Figure 2). Additionally, a random fragment of the *tetA* gene (721 bp, the mean length of all inserted genes) was cloned without a promoter into the pZS* plasmid and electroporated in *E. coli* to be used as the “wild type” strain (Acar Kirit et al., 2020, 2022). A copy of the 45 strains (44 *E. coli* genes and a “wild type”) was obtained to use in the present study. The insert size was confirmed by PCR on an agarose gel. The plasmids were then electroporated into *S.* Typhimurium 4/74 attλ::tetR-Kn^R^. Successful transformants were selected and after two rounds of streak purification on LB agar plates supplemented with 50 µg/mL ampicillin.

### Antibiotic and inducer concentrations

To determine the concentration of ciprofloxacin, growth rate measurements were carried out using Tecan Infinite 200 Pro Reader. For this, a 1:1,000 dilution of an overnight culture of *S.* Typhimurium strain 4/74 attλ::tetR-Kn^R^ carrying the pZS*-tetA (“wild type” plasmid) was grown in a 96-well plate in InSPI2 medium supplemented with different ciprofloxacin concentrations, and OD_600_ was measured every 20 min. The ciprofloxacin concentration used was that which decreased the growth rate in lnSPI2 by a half.

Gene expression on the plasmids was induced by addition of the inducer anhydrotetracycline (aTc)—a tetracycline derivative with no antibiotic activity—which interacts with TetR to induce gene expression (Ehrt et al., 2005). To determine the inducer concentration, *S.* Typhimurium strain 4/74 attλ::tetR-Kn^R^ was electroporated with a pZS* plasmid carrying a gfp gene under the control of the p_LtetO-1_ promoter. Relative fluorescence units were measured in InSPI2 with different inducer concentrations using Tecan Infinite 200 Pro Reader (excitation wavelength, 485 nm; emission wavelength, 535 nm). An arbitrary aTc concentration within the midlinear range of RFU curve was considered the optimal inducer concentration.

To understand the role of protein dosage as a potential barrier to HGT, the transferred genes on the plasmids were induced using increasing inducer concentrations thereby leading to increased protein levels in the cell (Acar Kirit et al., 2020). The inducer concentrations used were as follows: aTc 0 ng/mL (control), aTc 2 ng/mL (below optimal expression), aTc 4 ng/mL (optimal expression), aTc 6 ng/mL (above optimal expression), and aTc 8 ng/mL (saturation).

### Intrinsic properties of orthologs and statistical analysis

Effect of protein dosage was studied using growth rates of individual transformants relative to the “wild type.” A detailed protocol for the setup and measurement of growth rates is provided in Supplementary Methods. Growth rates for all transformants across all inducer concentrations were estimated from the log-linear part of the growth curve through regression analysis. Relative fitness (*w*) for the transferred genes was calculated by normalizing the growth rates of the transformants with the “wild type” for the respective inducer concentrations.

To categorize genes based on dosage effect, Mandel’s test for linearity was applied to the relative fitness values for every gene to decide whether the data points best fit a linear function than a quadratic function. These data used for Mandel’s test are provided in Supplementary Table 3. The test assesses if the residual variances of the linear and quadratic model are significantly different using the Fisher–Snedecor *F*-test (Andrade & Gómez-Carracedo, 2013; Brüggemann et al., 2006). The distribution of fitness data used to assess the effect of protein dosage is provided in Supplementary Table 4.


$$\begin{array}{l} F_{test}=((N-2){S_{y/x,lin}}^{2}-(N-3){S_{y/x,non}}^{2})/{S_{y/x,non}}^{2} \end{array}$$ (1)

In the above formula, *N* denotes sample size and *S* denotes the standard error of regression for the straight line (lin) and for quadratic (non)models.

Other selective factors affecting the DFEs of the transferred genes were studied using the NGS approach. The setup for the pooled growth assays, sequencing, and data processing is provided in Supplementary Methods. Each replicate pooled growth assay was normalized by the read depth of the vector backbone to account for variation in sequencing library preparation. Fitness costs of selected genes were estimated for each replicate by using the following regression model.


$$\begin{array}{l} \ln(1+s)=(\ln R_{t}-\ln R_{0})\;/\;t \end{array}$$

where *R*_0_ and *R*_t_ are the ratios of the frequencies of mutant (depth of gene) to “wild type” (depth of a nonfunctional tetA fragment) at the start and end points of the experiment, respectively, and t is the number of generations (Elena et al., 1998). The number of generations was estimated based on the dilution of the pooled stock by volume used during inoculation (100-mL media inoculated with 500-µL pooled stock, i.e., 200× dilution; number of generations = log_2_ 200 = 7.6 generations for all environments, except InSPI2 Hypoxic, for which the number of generations was 6.6). Forty-three genes were included in the analysis, as no reads were detected for one of the transferred genes (b1084).

To test whether the genes were neutral or not, a one-tailed one-sample *t*-test was applied on four replicate measurements for each gene, with µ_0_ < 1 or µ_0_ > 1. The *p*-values obtained were corrected for multiple tests using the FDR method (Benjamini & Hochberg, 1995), and α = 0.05 was used as the level of significance.

The transferred orthologs were divided into two groups using the Database for Clusters of Orthologous Groups (COGs). A one-sided Wilcoxon rank sum test was applied to the mean fitness values of genes for four replicate measurements to analyze whether the fitness effects of informational genes were smaller than those of the operational genes as predicted a priori (Jain et al., 1999; Nakamura et al., 2004; Rivera et al., 1998).

Additionally, we also looked at other intrinsic gene properties: the number of protein–protein interactions (PPI), metabolic interactions (MI), regulatory interactions (RI), gene length, GC content, and codon usage. The number of PPI, MI, and RI for the transferred orthologs in *S*. Typhimurium strain 4/74 was identified using the SalmoNet database (http://salmonet.org/). Gene length was calculated as the number of nucleotides in the coding sequence (CDS; i.e., from the start to the stop codon) of the transferred *E. coli* genes. GC content was calculated as the absolute deviation between the transferred *E. coli* genes and their corresponding *S.* Typhimurium strain 4/74 orthologs. Codon usage was estimated as the absolute deviation of the frequency of optimal codon usage (FOP) in the transferred *E. coli* genes using the FOP in *S.* Typhimurium (http://www.kazusa.or.jp/codon/cgi-bin/showcodon.cgi?species=602). Multiple linear regression was used to study the relationship between the intrinsic gene properties and fitness effects, by using the following model.


$$\begin{array}{l} Mean\;relative\;f\;\;itness\;\;(w)\;∼\;PPI+MI+RI+Gene\;length\;(bp)\\ +Deviation\;in\;GC\;content+Deviation\;in\;FOP\; \end{array}$$

Data used in the model are provided in Supplementary Table 5. The *p*-values obtained were corrected for multiple tests using the FDR method (Benjamini & Hochberg, 1995).

To investigate the effect of the environments on the central tendency of the DFEs, we used a paired and two-sided Wilcoxon signed rank test on each pairwise comparison of the four environments. To test whether the shape and spread of the distributions were significantly different between environments, a two-sided Kolmogorov–Smirnov (K–S) test was used. For each of these tests, the fitness values for genes were provided as a mean of four replicate measurements. The *p*-values obtained were corrected for multiple tests using the FDR method (Benjamini & Hochberg, 1995).

The gene × environment (G × E) interactions were studied using a two-way analysis of variance (ANOVA) test for each pairwise comparison for the four environments, using the following model.


$$\begin{array}{l} Relative\;\;\;f\;itness\;\;\;(w)\;\;∼\;Environment\;\times \;Gene\;\;\; \end{array}$$

The *p*-values obtained were corrected for multiple tests using the FDR method (Benjamini & Hochberg, 1995).

All statistical analyses were carried out using the R software (Version 4.0.2) in RStudio (Version 1.1.456).

## Results

*Salmonella* Typhimurium and *E. coli* were chosen due to their genetic and ecological similarity (Mugnai et al., 2015; Winfield & Groisman, 2003). Furthermore, the genetic similarity between the donor and recipient species ensures the functioning of the transferred genes in the host background, as well as that the functional interacting partners exist for most genes transferred; this is important as the study aims to understand the role of gene categories and gene interactions in the presence of horizontally acquired genes. Additionally, the donor and recipient are sufficiently divergent, so that the effect of several intrinsic factors of the transferred genes can be evaluated, such as their GC content and length.

### DFEs and role of other barriers to HGT

#### Estimating the DFEs of horizontally transferred genes

**Figure 1.**
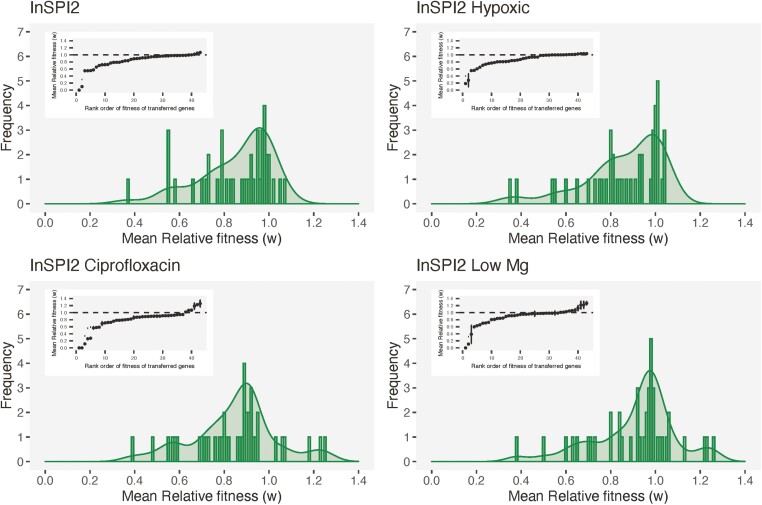
Distribution of fitness effects of transferred *Escherichia coli* orthologs expressed in *Salmonella* Typhimurium 4/74. The figure shows the distribution of fitness effects obtained from pooled growth assays in each infection-relevant environment. On the x-axis, transferred genes are ordered by their relative fitness values in the respective environments. Error bars represent *SD* of the mean for four replicate measurements for each gene. Embedded plots are histogram and density representations for the same data. Black dashed line shows a fitness of 1.

In line with the previously observed DFEs (Elena et al., 1998; Eyre-Walker & Keightley, 2007; Sanjuan et al., 2004), the fitness effects of our 44 genes in 4 different environments are best explained by a log-normal distribution (µ_InSPI2_ = −0.211, σ_InSPI2_ = 0.243; µ_InSPI2 Hypoxic_ = −0.174, σ_InSPI2 Hypoxic_ = 0.200; µ_InSPI2 Ciprofloxacin_ = −0.262, σ_InSPI2 Ciprofloxacin_ = 0.308; µ_InSPI2 Low Mg_ = −0.148, σ_InSPI2 Low Mg_ = 0.252; Figure 1). Estimated parameters for other compatible distributions are provided in Supplementary Table 6.

### Categorizing genes based on fitness effects

**Figure 2.**
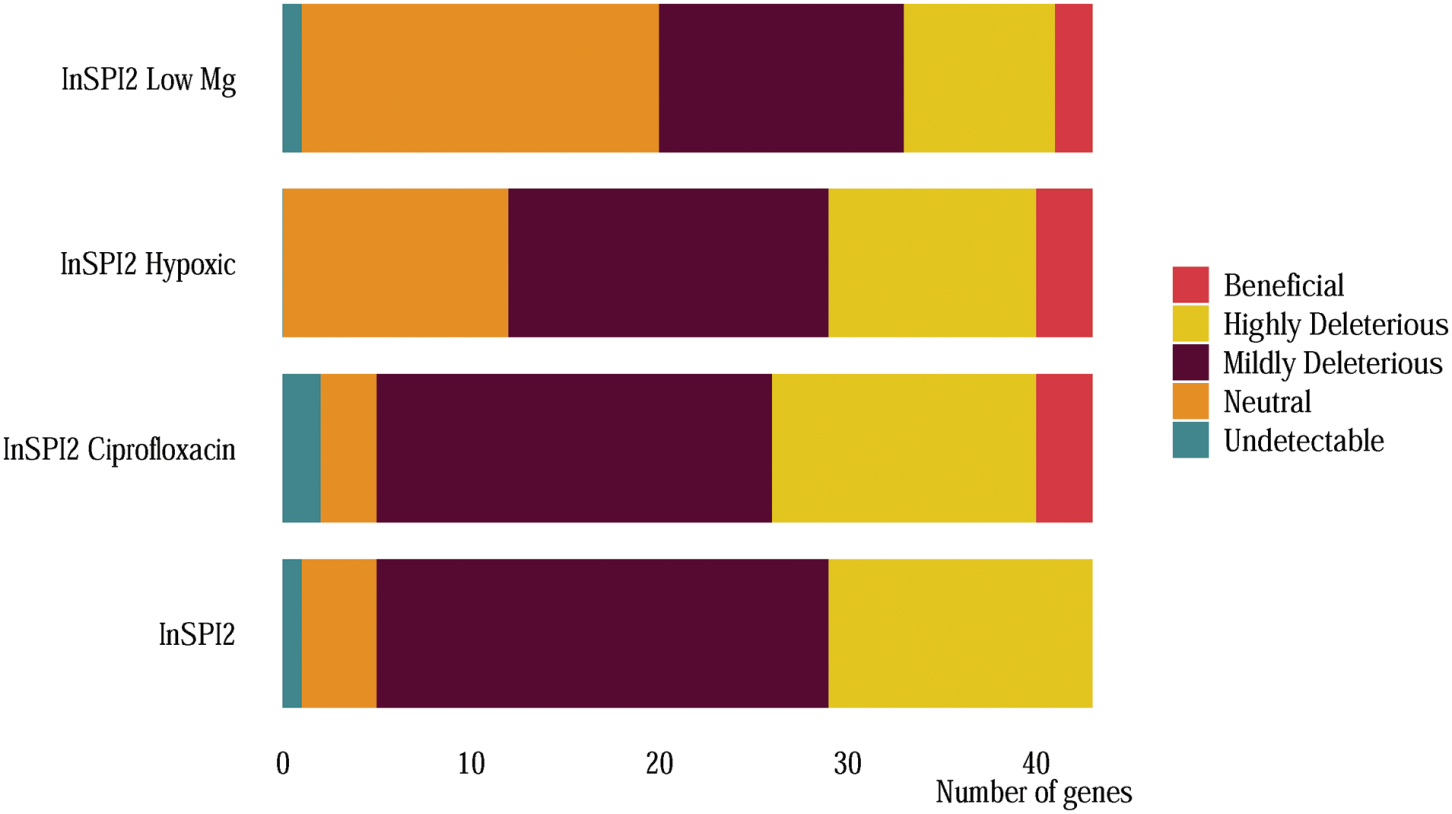
Grouping of genes based on fitness effects. Breakdown of the transferred orthologs based on results from one-sample *t*-test in each growth environment. Undetectable genes: mean fitness of gene = 0, *p*_adj_ < .05 with µ_0_ < 1; neutral genes: *p*_adj_ > .05 with µ_0_ > 1 and µ_0_ < 1; mildly deleterious genes: 1 > mean fitness of gene > 0.8, *p*_adj_ < .05 with µ_0_ < 1; highly deleterious genes: mean fitness of gene < 0.8, *p*_adj_ < .05 with µ_0_ < 1; beneficial genes: *p*_adj_ < .05, with µ_0_ > 1.

Based on the results from the one-sample *t*-test, the genes were categorized into five groups in each environment with respect to their fitness effects (Figure 2). On average across the four environments ~70% of the genes were deleterious, although ~43% of these imposed a relatively minor fitness cost (mildly deleterious), whereas ~27% were highly deleterious. Approximately 22% of the genes were found to be neutral on average across the four environments (Figure 2). FDR-corrected *p*-values for fitness effects of *E. coli* orthologs deviating from neutrality are provided in Supplementary Table 7.

Comparing between the environments, most genes were found to impose a deleterious fitness effect in the InSPI2 environment (38 of 43), followed by InSPI2 Ciprofloxacin (35 of 43), InSPI2 Hypoxic (28 of 43), and InSPI2 Low Mg (21 of 43). Additionally, InSPI2 was the only environment not having any genes showing a beneficial effect (Figure 2).

### Functional category of genes

We separated our transferred genes based on their COG annotations as informational (19 genes) and operational (20 genes) and tested whether or not the functional categories influenced the fitness effects of the transferred genes (excluding the genes that have not been classified [3 genes] and those that were classified as both informational and operational [2 genes]).

**Figure 3.**
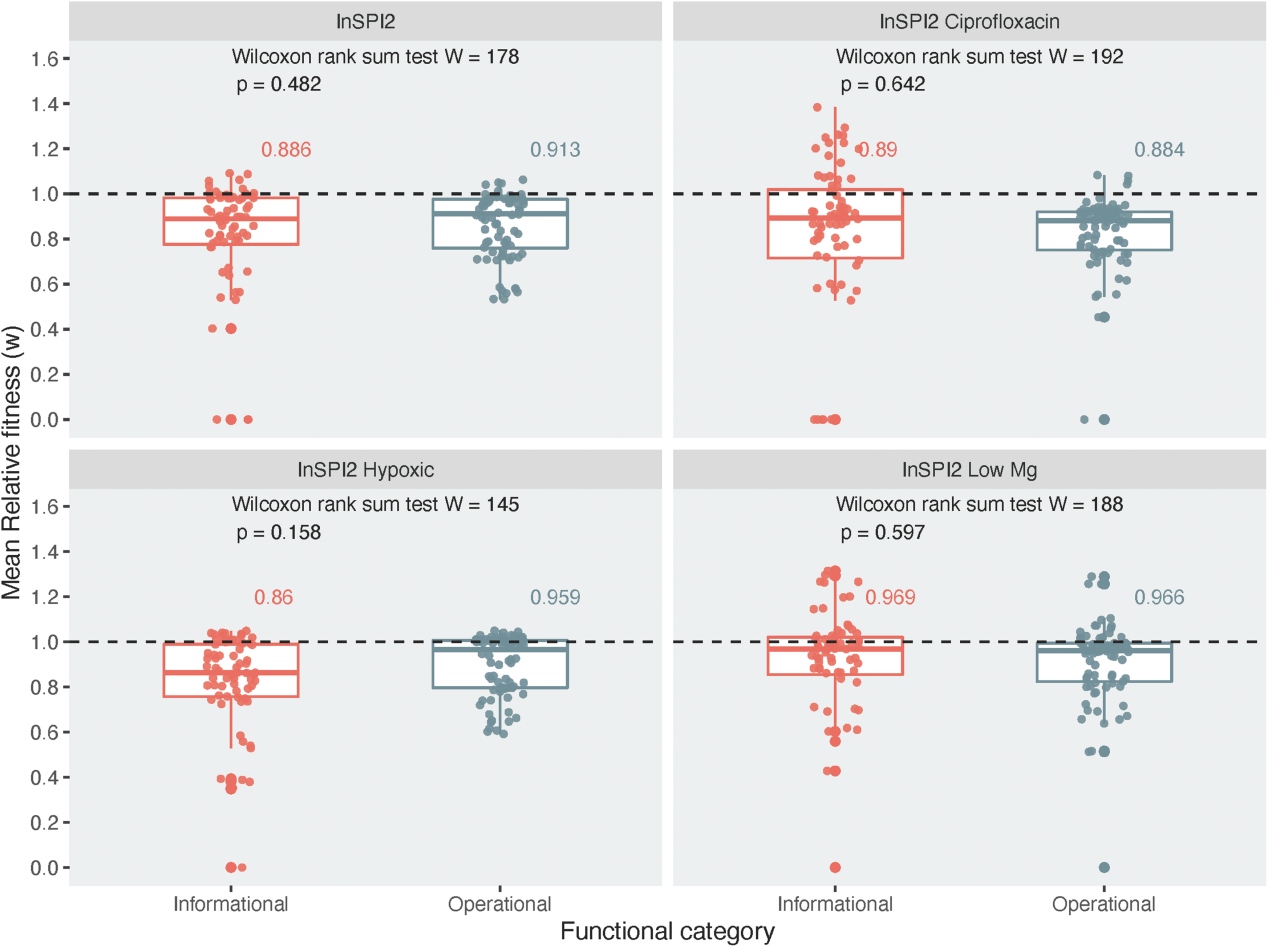
Fitness effects by functional categories. Boxplot with jitter points showing the fitness effects of transferred genes (four replicate measurements) in four growth environments, with genes divided into two functional categories. Black dashed line shows a fitness of 1.

Contrary to the complexity hypothesis, we did not observe a significant difference in the mean fitness effects of genes between the two functional categories, across all growth environments (Figure 3). Additionally, we further categorized the genes based on their functional annotations to investigate the effect of specific functions on fitness. We did not find a significant difference in the mean fitness effects of genes between the functional classes (Wilcoxon rank sum test, Supplementary Table 8, Supplementary Figure 6). An extended table for all the genes is provided in Supplementary Table 9.

### Complexity of gene interactions

**Figure 4.**
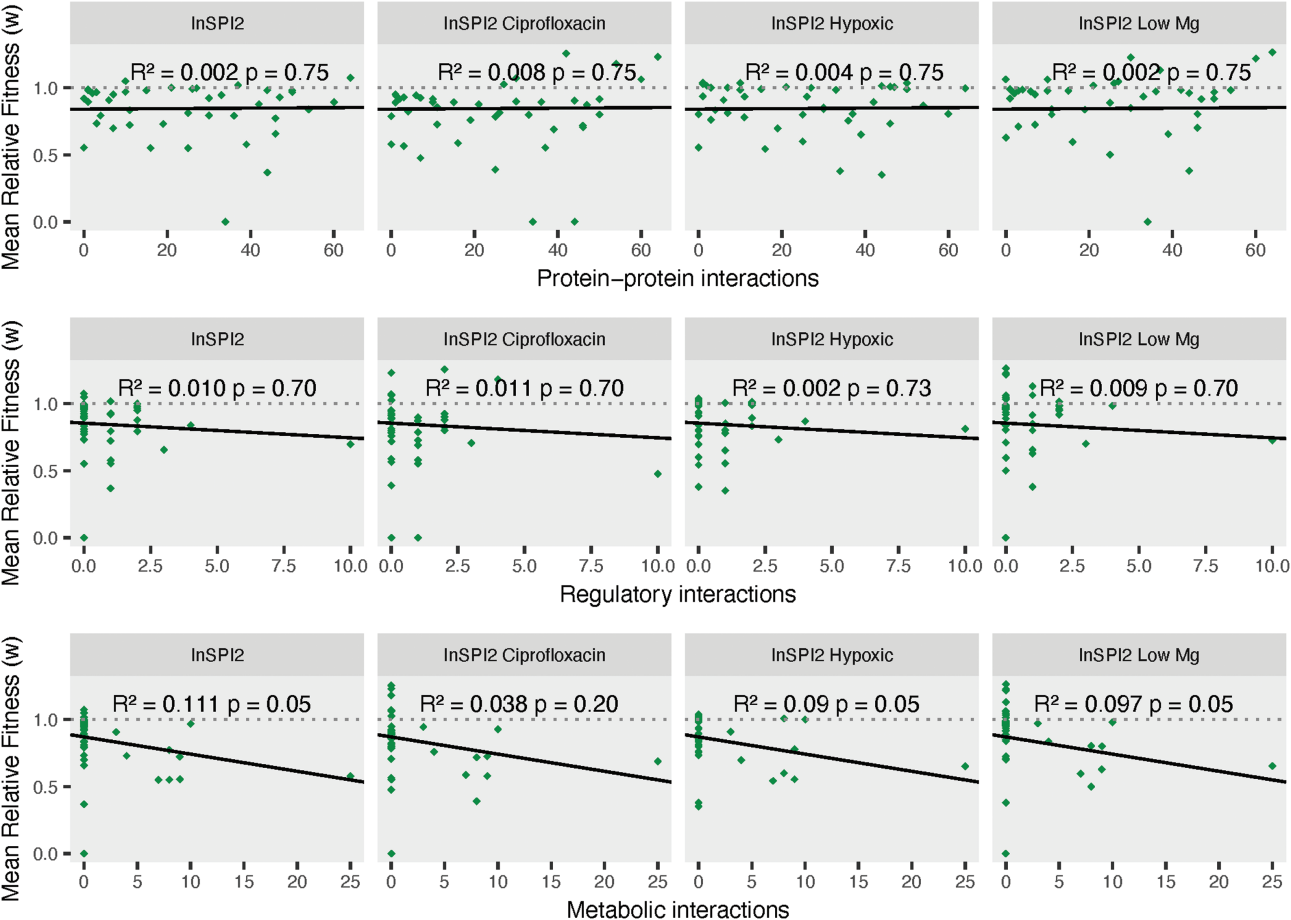
Relationship between relative fitness and number of interactions. Relative fitness of transferred *Escherichia coli* orthologs in four growth environments plotted against the number of protein–protein interactions (A), regulatory interactions (B), and metabolic interactions (C) in *Salmonella* Typhimurium 4/74. The black line is the regression between the two variables. Gray dotted line shows a fitness of 1. FDR-corrected *p*-values and *R*^2^ values from the linear regression analysis are shown on the plot.

To test whether high gene connectivity restricts HGT events, we investigated the relationship between fitness of the transferred orthologs and their corresponding interactions at three levels (protein–protein, metabolic, and regulatory) in *S.* Typhimurium 4/74. None of the gene interaction levels could significantly explain the fitness effects of the transferred orthologs in the four environments (Figure 4).

There might be a potential trend for increasing number of MI leading to reduced fitness of *S.* Typhimurium 4/74 (Figure 4C). However, based on a power analysis (Cohen, 1992), the likelihood of the regression model to detect a significant trend was found to be 62%, suggesting that an increased number of high-complexity genes might be needed to identify the effect of increasing metabolic interactions on HGT.

### GC content and codon usage

The effects of GC content and codon usage on fitness were examined, although these measures are highly correlated (Sueoka, 1961). We did not observe a significant relationship between the absolute deviation in GC content between the gene orthologs and the observed fitness effects in four growth environments (Supplementary Figure 5a). Similarly, the absolute deviation in the frequency of optimal codon usage (FOP) between the transferred and endogenous gene copies could not explain the observed fitness effects in any environment (Supplementary Figure 5b).

### Gene length is a significant predictor of HGT

**Figure 5.**
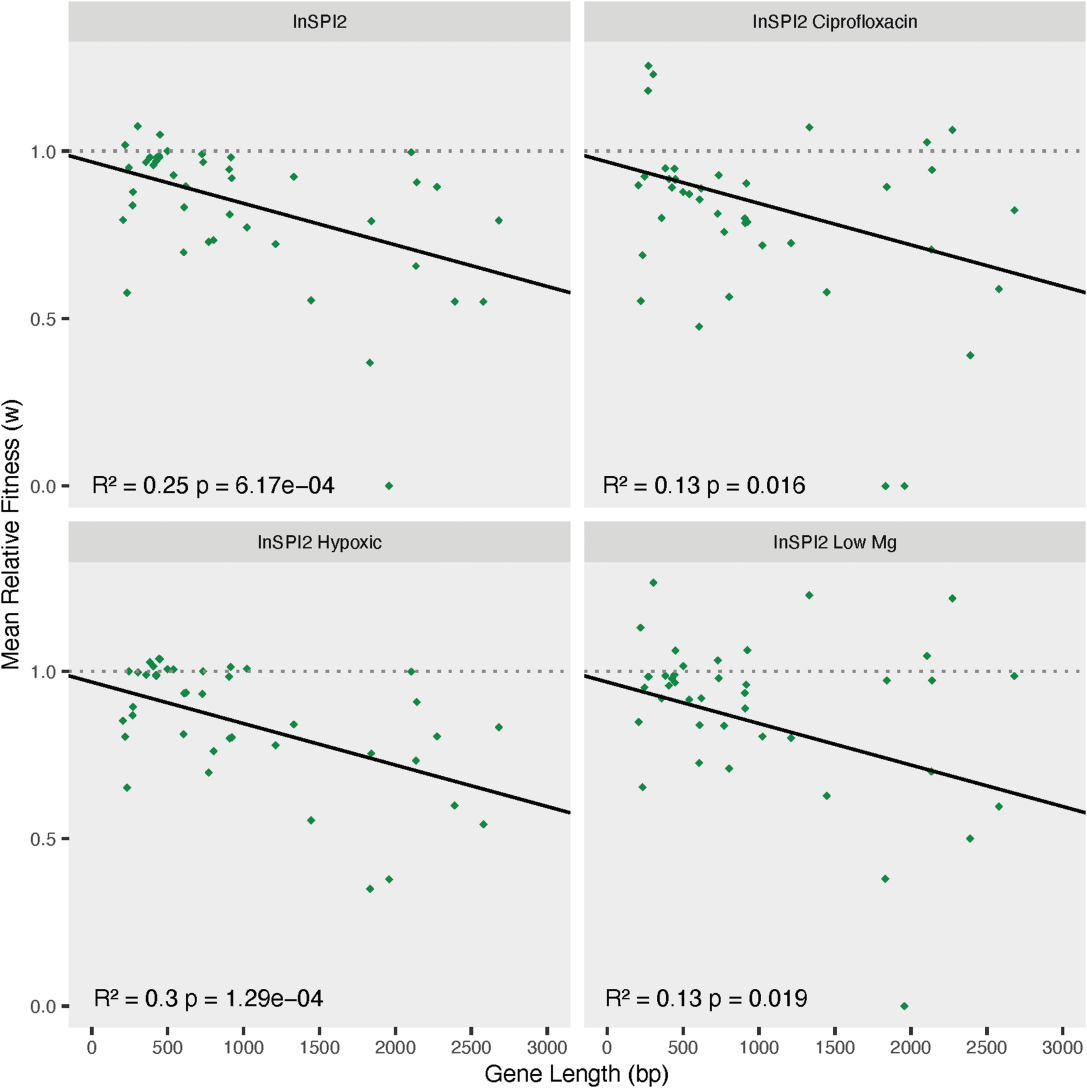
Relationship between relative fitness and gene length. Relative fitness of transferred *Escherichia coli* orthologs in *Salmonella* Typhimurium 4/74 plotted against the gene length in four growth environments. The black line is the regression between the two variables. Gray dotted line shows a fitness of 1. FDR-corrected *p*-values and *R*^2^ values are shown on the plot.

We observed a statistically significant negative relationship between gene length and the fitness effects of the transferred genes in all growth environments (Figure 5).

### Environment shapes the trajectory of HGT

The impact of the environment on the fitness effects of genes has been well documented in studies of antibiotic resistance genes and metabolic enzymes (Melnyk et al., 2015; Zhong et al., 2020). Thus, we posed the following questions: Do environments affect the central tendency, shape, or spread of the DFEs? Do environments differentially affect the fitness effects of different genes? The DFEs are fundamental in predicting the response of a population to selection, as the shape of the DFEs can provide an estimation of the effect of a new gene transfer event or mutation (Eyre-Walker & Keightley, 2007).

We observed significant differences in median fitness effects for InSPI2 Hypoxic and InSPI2 Low Mg were observed in comparison with InSPI2. Also, the median fitness effects for InSPI2 Ciprofloxacin and InSPI2 Low Mg were significantly different from InSPI2 Hypoxic and InSPI2 Ciprofloxacin, respectively (Wilcoxon rank sum test, Supplementary Table 10a, Supplementary Figure 7). The shape and spread of DFE for InSPI2 Low Mg were found to be significantly different from InSPI2 Ciprofloxacin (Kolmogorov–Smirnov test, Supplementary Table 10b). This potentially indicates that certain environments can significantly affect the shape and spread of fitness effects after HGT.

**Figure 6.**
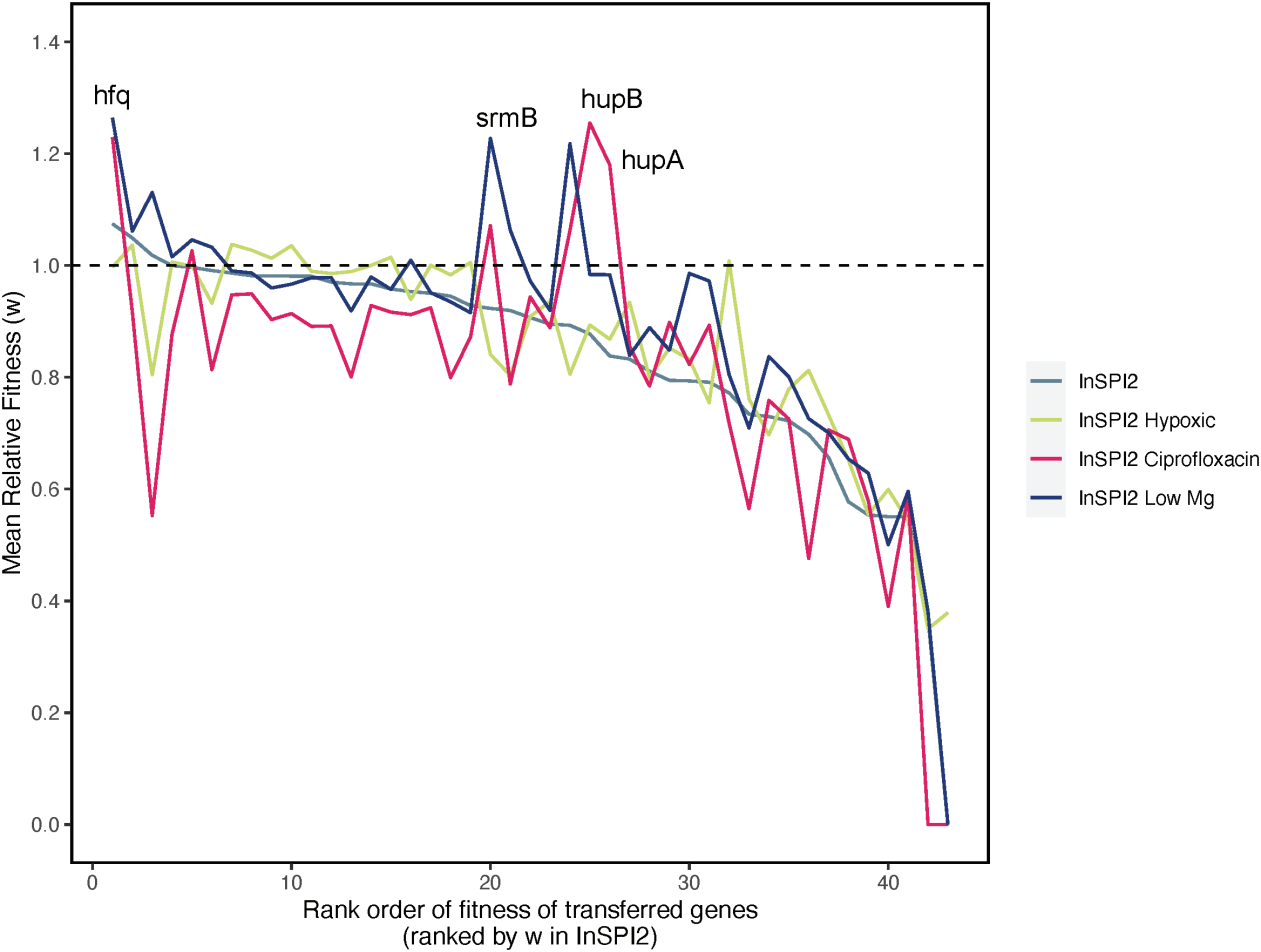
Relative fitness of transferred genes in four environments. The figure shows the variation in fitness effects of individual genes between environments, few examples have been labeled. Genes are ranked according to the relative fitness measurements in the control environment (InSPI2) only. Black dashed line shows a fitness of 1.

Additionally, we observed a strong interaction between individual genes and the environment (*F*_126,516_ = 13.32, *p* < 0.001), suggesting that the environments interact with genes differentially (Figure 6). We observed 29 of 43 genes showing significant gene × environment (G × E) interactions (see Supplementary Table 11). This indicates that the success of HGT events is largely dominated by specific G × E interactions.

### Protein dosage is a barrier to HGT

**Figure 7.**
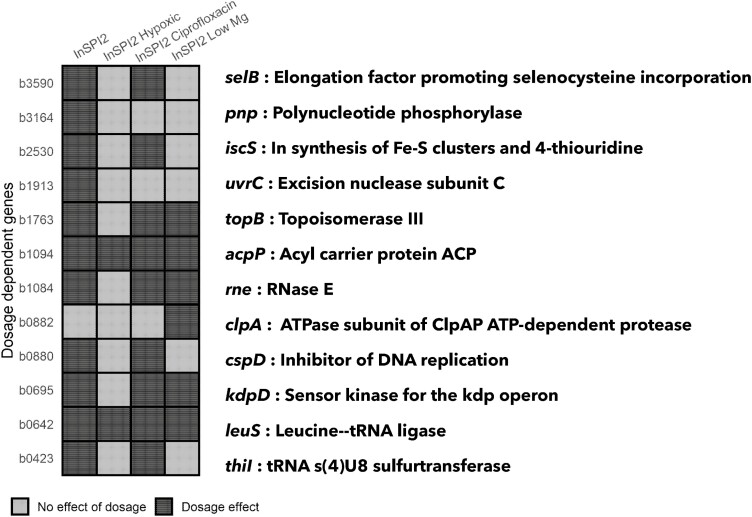
Dosage-dependent genes. The figure shows *Escherichia coli* orthologs (on y-axis) that exhibit a dosage effect across different environments (on x-axis) when expressed in *Salmonella* Typhimurium 4/74. Gene names and functions are mentioned on the right.

Due to the genetic similarity of *E. coli* and *Salmonella* (McClelland et al., 2001), observed fitness effects due to increase in gene expression can be attributed to increased protein levels in the cell (i.e., a dosage effect). To this end, we applied Mandel’s test on the fitness values obtained and identified 12 genes showing a dosage-dependent response (see Supplementary Table 12; Figure 7).

The number of dosage-dependent genes was found to be highest in the InSPI2 environment (11 of 44 genes), followed by InSPI2 Ciprofloxacin (9 of 44 genes) and InSPI2 Low Mg (6 of 44 genes) and was lowest in the InSPI2 Hypoxic environment (2 of 44 genes). In summary, 9 of the 44 transferred genes exhibited dosage dependence in a minimum of two growth environments (Figure 7). Classification of all genes based on results from Mandel’s test across all environments is provided in Supplementary Table 12.

## Discussion

Despite studies suggesting rampant uptake of foreign genetic material, certain selective forces can act as barriers to HGT (Baltrus, 2013; Gonzalez-Candelas & Francino, 2012; Popa & Dagan, 2011). This study sets out to gain a quantitative understanding of the intrinsic properties of the transferred genes that prevent their successful movement from one species to another. In a previous study, we highlighted the importance of conducting experimental fitness measurements in natural environments (Acar Kirit et al., 2020, 2022), which are scarce due to experimental limitations and their labor-intensive nature. Our study addresses this paucity, by conducting fitness assays in growth environments that capture the conditions encountered by *S.* Typhimurium 4/74 during an intracellular infection (Kroger et al., 2013).

Variations in plasmid copy number and gene expression levels can create noise in fitness estimations. To circumvent this, our study uses a low copy plasmid system, where the promoters driving gene expression are tightly repressible, and the variance in plasmid copy numbers is low (Lutz & Bujard, 1997).

Fitness is primarily evaluated using bacterial growth rates (Bennett et al., 1990; Lenski et al., 1994, 1998). The density of the microbial culture is directly proportional to the optical density (OD); however, this is only true for a limited OD range, above which measuring culture density with high precision can be demanding (Mira et al., 2022). Additionally, external factors affecting the morphology of bacterial cells, such as filamentation, elongation, or clumping of cells can interfere with OD measurements, thereby affecting growth rate measurements (Sridhar et al., 2021; Vadia & Levin, 2015). Although there were limitations to the use of OD for fitness estimations, the approach allowed us to test fitness effects across different levels of gene expression from no expression to saturation and to understand the effect of protein dosage in a high-throughput and cost-effective manner.

Furthermore, we investigated the role of intrinsic properties of the transferred genes and the environment as potential barriers to HGT using NGS. To do this, we pooled the 44 transformants in equal ratios and grew them continuously for approximately eight generations in the four infection-relevant environments, and estimated fitness from changes in gene frequencies. The rationale behind pooling of transformants being the approach is time and cost effective, allows for a greater number of replicate experiments, and is less labor intensive. Additionally, studies have shown that copy numbers of a gene are exponential functions of growth rate (Bremer & Dennis, 2008; Klumpp et al., 2009; Roller et al., 2016).

Since the aim of the study was to understand what affects the presence of a newly acquired gene in a population, we tested the immediate fitness effects after a new gene is expressed in the recipient. Although performing pooled growth experiments for a longer duration would help understand the dynamics between transformants due to constrained resources, it could also potentially lead to beneficial compensatory mutations that would balance out the effects of the newly transferred gene. The short duration of the experiment ensured that the energy source was not depleted thereby indicating that the transformants were not competing intensely against each other, and the fitness differences were mainly due to differences in the growth rates of transformants.

Although computational approaches have the benefit of using a large sample size to infer the evolutionary outcomes of HGT, discrepancies in the method of analysis and their underlying assumptions can result in conflicting conclusions. Additionally, these approaches cannot provide information on the role of the environment at the time of the transfer event. This study provides a truly systematic quantitative understanding of the presence of horizontally transferred genes, and the evolutionary barriers to the emergence and evolution of novel phenotypes. The current study did not observe gene interactions, GC content, and functional category of the genes as significant factors hindering the success of HGT. These observations are in congruence with previous results (Acar Kirit et al., 2020, 2022), suggesting that these barriers are either only weak forces or are below the threshold of practical measurements, especially when the donor and recipient species have diverged recently. Alternatively, the exclusion of mobile genetic elements, and the lack of representative interacting partners for MI and RI, creates a bias in the gene set selected for this study and might have an impact on the inference of the effect of these barriers.

However, gene length was found to have a significant negative fitness effect on the presence of horizontally acquired genes. This effect of gene length might be potentially explained by ribosomal sequestration (Shah et al., 2013). An alternate explanation could be the intrinsically disordered protein regions lacking a tertiary structure, which have been found to be correlated with gene length (Acar Kirit et al., 2020).

There has been limited support from computational studies for the effects of protein dosage as an evolutionary barrier to HGT mainly because it is difficult to infer dosage levels from genomic data. Our data provide evidence for protein dosage to be a significant barrier to HGT with 12 genes being identified as dosage dependent. Of the 12 dosage-dependent genes, 11 genes were found to be natively expressed at low levels in *S.* Typhimurium 4/74 (Kroger et al., 2013). Low levels of native expression potentially suggest that these genes might be maintained by *Salmonella* as single copy and any increase in their protein concentrations potentially imposes a significant fitness cost in the recipient. Interestingly, genes showing a dosage effect were found to be heavily influenced by the environment (Figure 7). Additionally, toxicity due to increased gene expression was also found to be a barrier to HGT (see Supplementary Table 4). A possible mechanism for toxicity effects could be attributed to the poorly optimized gene interactions due to the lack of coevolution between orthologs or promiscuous protein interactions.

Although the importance of the environment has been implicated in the gene acquisition process, we lack an understanding of how the environment determines if the acquired gene is likely to be maintained following HGT. Therefore, analyzing the DFE of horizontally transferred genes across different environments is essential to understand if HGT is an opportunistic process or is primarily determined by the intrinsic properties of the newly acquired gene. Our data show strong G × E interactions, with the fitness effects of the gene becoming unpredictable in each environment. In some cases, the specific function of the gene might be able to explain the observed fitness effect in a certain environment. For example, the *hfq* gene was beneficial for *S.* Typhimurium 4/74 in InSPI2 Ciprofloxacin and InSPI2 Low Mg environments (Figure 6). The *hfq* gene is an RNA chaperone facilitating transcriptional regulation in response to environment stress and changes in metabolite concentrations (Guisbert et al., 2007). Additionally, *hfq* has been shown to regulate the formation of persister cells through the MqsR toxin in *E. coli* (Kim & Wood, 2010). Similarly, *srmB* gene was beneficial for *S.* Typhimurium 4/74 in InSPI2 Ciprofloxacin but had a neutral effect on the host in InSPI2 Low Mg (Figure 6). The *srmB* gene is a DEAD-box RNA helicase facilitating ribosomal assembly (Rabuck-Gibbons et al., 2020). Additionally, it has been shown that SrmB belongs to the AMR Gene Family—ABC-F ATP-binding cassette ribosomal protection proteins, which confer antibiotic resistance via ribosomal protection, unlike other ABC proteins that confer resistance through antibiotic efflux (Alcock et al., 2020; Murina et al., 2019; Sharkey et al., 2016; Yang et al., 2022). However, since ciprofloxacin is known to inhibit DNA replication, the *srmB* gene might have a potential indirect role in regulating fluoroquinolone resistance intracellularly. This suggests that improving our knowledge of gene functions could potentially support our understanding of patterns of HGT.

An area of the study that could be expanded upon would be estimating fitness effects of the transformants in macrophages. Even so, the current data represent the closest distribution of fitness effects of horizontally transferred genes achievable using an in vitro model. Our data show which of the environmental variables of a heterogeneous intracellular environment might be favoring HGT, highlighting that the environmental effects should be accounted for when making predictions about the outcome of HGT events. The approach outlined in this study can be further used to study the DFE of genes transferred between phylogenetically distant species to understand which properties of the gene favor or restrict the success of transfer. For example, transferring orthologs from *E. coli* to *Lactobacillus* certain barriers such as GC content or gene interactions might impose significant fitness costs on the recipient thereby hampering the presence of the newly acquired gene. The fitness costs could arise due to disruption of functional interactions on the introduction of a highly divergent partner or that the divergent genes may be involved in novel negative interactions and that the recipient genome has not evolved to buffer these deleterious side effects.

In conclusion, our data provide evidence that the environment plays a crucial role in influencing the magnitude of gene length and protein dosage as significant evolutionary barriers to the success of gene transfer events.

## Supplementary material

Supplementary material is available online at *Evolution Letters* (https://academic.oup.com/evlett/qrad020).

qrad020_suppl_Supplementary_Tables

qrad020_suppl_Supplementary_Material

## Data Availability

The raw data for the pooled growth sequencing experiment are publicly available. The unmerged reads and the read depth files are deposited in NCBI GEO repository with accession number GSE223644 (https://www.ncbi.nlm.nih.gov/geo/query/acc.cgi?acc=GSE223644). The merged reads are deposited in Mendeley Data and can be accessed at https://data.mendeley.com/datasets/44dtg2m6y9/1. The codes for all analysis and plots have been made available on GitHub (https://github.com/ramabht/Barriers_to_HGT).
