# Supplementary material for: Evolutionary barriers to horizontal gene transfer in macrophage-associated Salmonella
Source: Evol Lett. 2023 May 23;7(4):227–39. doi: 10.1093/evlett/qrad020 (PMC10355182; doi:10.1093/evlett/qrad020)
Supplement: qrad020_suppl_Supplementary_Material [file qrad020_suppl_supplementary_material.docx]

**Supplementary Methods**

**Growth measurements using Growth Profiler 960**

A 1:20 dilution of individual overnight cultures of the 44 S. Typhimurium 4/74 attλ::tetR-Kn^R^, pZS* carrying transformants were grown individually in InSPI2 medium supplemented with 50 µg/ml Ampicillin to OD_600_ 0.2. The position of the strains was randomised on 96 well plates to minimise position effects, with each starter plate carrying two technical replicates for each of the 44 transformants and ‘wild-type’. For each environment, cultivations were carried out in 96-half-deepwell microplates (CR1496dg, Enzyscreen) with gas-permeable sandwich covers and clamps, except InSPI2 Hypoxic, where cultures were overlaid with sterile paraffin oil and gas-permeable sandwich covers were used in conjunction with carbon-flush clamp units. A total volume of 250µl including a 15µl inoculum from the starter plates with randomised layout of genes was used throughout the experiment. The wells were additionally supplemented with different aTc concentrations to induce gene expression (see Antibiotic and Inducer Concentrations above). Supplementary Figure 3 shows a graphical representation of the methodology. Each experiment was repeated to produce 4 biological replicates across two days. Plates were incubated for 18 hours at 37℃ and shaken at 250 rpm, except for InSPI2 Hypoxic, where cultures were grown statically. Measurements were recorded every 20 mins using optimal image scanning by Growth Profiler 960 platform (Enzyscreen). The green values (G-values) obtained for each well were converted into their equivalent OD_600_ values using the image analysis software GP960Viewer (Enzyscreen).

**Pooled Growth Assays**

A 1:10 dilution of individual overnight cultures of the 44 S. Typhimurium 4/74 attλ::tetR-Kn^R^, pZS* carrying transformants were grown in InSPI2 medium supplemented with 50 µg/ml Ampicillin to OD_600_ 0.2. The following steps were carried out on ice. After reaching the target OD_600_, strains were mixed at equal volumes to make a total of 135mL. The pooled stock was thoroughly mixed and, 50 aliquots - each containing 1 ml pooled stock and 250µl 80% glycerol, were stored at -80℃. The remaining volume of the pooled stock was split to create two technical replicates for the starting frequency of the pooled growth experiments. To verify the cell concentration of the pooled stock, serial dilutions of a frozen stock were plated on LB agar plates supplemented with 50 µg/ml Ampicillin to obtain CFU counts. The pooled stock concentration was approximately 5 x 10^7^ CFU/ml.

To perform the pooled growth experiments, frozen aliquots of the pooled stock were thawed on ice, and then gently mixed by pipetting. A total volume of 100ml including an inoculum of 10^7^ cells from the pooled stock was used throughout the experiment. Gene expression was induced by the addition of 4ng/ml aTc, and 50 µg/ml Ampicillin was used for maintenance of pZS* plasmids carrying the transferred genes. For each of the four infection relevant environments (Supplementary Table 1), 100ml volume of cultures were grown in 200ml conical flasks, except InSPI2 Hypoxic, where cultures were filled up to the brim of 50ml conical tubes, capped with rubber septum, and further sealed using parafilm. The flasks were incubated at 37℃ and 250 rpm and grown to OD_600_ 0.4. For the InSPI2 Hypoxic environment, cultures were grown on static at 37℃ to OD_600_ 0.2. Four replicate experiments were performed for each of the environments under study. On reaching the target OD (i.e., endpoint of the experiment), plasmid DNA was extracted from the total culture volume using Invitrogen^TM^ PureLink^TM^ HiPure Plasmid Midiprep Kit (Catalog number: K210004). Supplementary Figure 4 provides a graphical illustration of the pooled growth setup.

**Sequencing**

DNA libraries for 16 biological replicates (4 replicate pooled experiments for each of the 4 environments) and 2 technical replicates (starting frequency of pooled growth experiments) were prepared and sequenced on Illumina NovaSeq 6000 platform (PE150bp) by our collaborators at the Laboratories of Molecular Anthropology and Microbiome Research (LMAMR), University of Oklahoma, USA, resulting in ~3.2 million read pairs per sample. The distribution of the raw data is provided as **Supplementary Table 2**.

To briefly describe the library preparation protocol, DNA was first fragmented by sonication using Qsonica 800R, followed by construction for Illumina compatible sequencing libraries using the Kapa Hyperprep kit following manufacturer’s protocols. Sequencing libraries were generated using PCR with Illumina-specific barcoded primers and dual index approach was used to allow multiplexing. The amplified library was then validated using 4150 TapeStation System (Agilent Technologies, Inc.), and size selected using Pippin Prep, BluePippin (Sage Science, Inc.). The libraries were pooled in equimolar concentrations and were sequenced.

**Processing of Sequencing Data**

Quality checks and demultiplexing of sequenced reads were performed by our collaborators. Sequenced reads were then processed using AdapterRemoval (v2) (Schubert et al. 2016) to trim regions with low quality bases (q<30), merge overlapping read pairs, remove Illumina adapter sequences, and remove reads containing ambiguous bases (Ns).

A customised reference genome consisting of FASTA format sequences of the 44 transferred E. coli orthologs, ‘wild type’ tetA fragment, pZS* plasmid backbone, complete sequences of S. Typhimurium 4/74 chromosome (GenBank: CP002487.1); and its 3 native plasmids TY474p1 (GenBank: CP002488.1), TY474p2 (GenBank: CP002489.1), and TY474p3 (GenBank: CP002490.1) was built using Bowtie2 (Version 2.4.4). The sequenced reads were then mapped to the custom-built reference genome using the defaults local alignment parameter in Bowtie2 (Version 2.4.4). The mapped SAM files were converted to BAM format and the corresponding BAM files were sorted using Samtools (Tools for Alignment in SAM format) (Version 1.13). Read depths at each nucleotide position were calculated using depth function of Samtools (Version 1.13). Nucleotide depths were converted to gene frequencies by calculating the median of depths obtained for every gene using the R software (Version 4.0.0). All steps of the sequencing data processing were performed on Ubuntu 16.04.5 LTS (GNU/Linux 4.15.0-52-generic x86_64).

**References**

Schubert, M., Lindgreen, S. & Orlando, L. 2016. AdapterRemoval v2: rapid adapter trimming, identification, and read merging. BMC Res Notes, 9, 88.

**Supplementary Figures**

**Supplementary Figure 1 Schematic representation of *Salmonella enterica* serovar Typhimurium attλ::tetR-KnR 4/74 strain**

Recipient strain for genes transferred from *Escherichia coli* K-12 MG1655.

**Supplementary Figure 2 Schematic representation of expression plasmid used to construct 4/74 mutant library**

The pZS* plasmid consisting of an SC101 origin of replication (blue), terminators (t0 and T1) (orange), Ampicillin resistance gene (pink), transferred E. coli gene (yellow) under the control of the inducible pLtetO1 promoter (green).

**Supplementary Figure 3 Graphical representation for preparation of mutant library and 96 well plates**

a. shows the workflow for construction of S. Typhimurium strain 4/74 mutant library. b. left shows 96 well starter plate preparation, yellow and blue colours showing the two replicates for 5 mutants and a ‘wild type’; and right shows 96 well plate preparation for growth measurements, shades of green from left to right showing increasing inducer concentrations.

**Supplementary Figure 4 Graphical representation for pooled growth experiments**

The flask-2 icon in the figure [by DCBLS 🔗](https://togotv.dbcls.jp/en/pics.html) is licensed under [CC-BY 4.0 Unported 🔗](https://creativecommons.org/licenses/by/4.0/) / Object properties modified from original. The genomesequencer9 icon [by DCBLS 🔗](https://togotv.dbcls.jp/en/pics.html) is licensed under [CC-BY 4.0 Unported 🔗](https://creativecommons.org/licenses/by/4.0/) .

**Supplementary Figure 5 Relationship of GC content and Codon Usage with fitness**

Relative fitness of transferred E. coli orthologs in S. Typhimurium 4/74 plotted against absolute deviation in GC Content (a) and absolute deviation in FOP (b) in four growth environments. The black line is the regression between the two variables. Grey dotted line shows a fitness of 1. FDR corrected p-values and R^2^ values from the linear regression analysis are shown on the plot.

**Supplementary Figure 6 Relationship of Gene function with fitness**

Relative fitness of transferred E. coli orthologs in S. Typhimurium 4/74 plotted against gene functions in four growth environments. Black dashed line shows a fitness of 1.

**Supplementary Figure 7 Fitness effects of transferred genes**

Bee swarm boxplot showing the distribution of fitness effects of transferred genes (4 replicate measurements) in four growth environments.

**Supplementary Tables (available as separate files)**

**Supplementary Table 1** – Infection relevant environments used in the study.

**Supplementary Table 2** – NGS read distribution for each sample.

**Supplementary Table 3** – Fitness data obtained using growth measurements.

**Supplementary Table 4** – Distribution of fitness data to assess effect of protein dosage.

**Supplementary Table 5** – Fitness data obtained from pooled growth assays.

**Supplementary Table 6** – Estimated parameters for compatible DFE models.

**Supplementary Table 7** – FDR corrected p-values for one-tailed one sample t-test.

**Supplementary Table 8** – Summary statistics to assess the relationship between fitness effects and functional classes of orthologs.

**Supplementary Table 9** – *E. coli* orthologs used in the study.

**Supplementary Table 10** – Summary statistics to assess the effect of environment on the properties of DFEs.

**Supplementary Table 11** – Genes showing significant G X E interactions.

**Supplementary Table 12** – Genes showing a dosage dependent response.
